# Development and validation of an ECM-related prognostic signature to predict the immune landscape of human hepatocellular carcinoma

**DOI:** 10.1186/s12885-022-10049-w

**Published:** 2022-10-04

**Authors:** Guozhi Wu, Yuan Yang, Rong Ye, Hanxun Yue, Huiyun Zhang, Taobi Huang, Min Liu, Ya Zheng, Yuping Wang, Yongning Zhou, Qinghong Guo

**Affiliations:** 1 The First Clinical Medical College, Lanzhou University, Lanzhou, 730000 Gansu China; 2 Department of Gastroenterology, The First Hospital of Lanzhou University, No.1 West Donggang Road, Lanzhou, 730000 Gansu China; 3 Gansu Key Laboratory of Gastroenterology, Lanzhou University, Lanzhou, 730000 Gansu China; 4 Department of Radiology, the First Hospital of Lanzhou University, Lanzhou, 730000 Gansu China

**Keywords:** Hepatocellular carcinoma, Extracellular matrix, LncRNA, Immune infiltration, Tumour mutation burden

## Abstract

**Background:**

The global burden of hepatocellular carcinoma (HCC) is increasing, negatively impacting social health and economies. The discovery of novel and valuable biomarkers for the early diagnosis and therapeutic guidance of HCC is urgently needed.

**Methods:**

Extracellular matrix (ECM)-related gene sets, transcriptome data and mutation profiles were downloaded from the Matrisome Project and The Cancer Genome Atlas (TCGA)-LIHC datasets. Coexpression analysis was initially performed with the aim of identifying ECM-related lncRNAs (*r* > 0.4, *p* < 0.001). The screened lncRNAs were subjected to univariate analysis to obtain a series of prognosis-related lncRNA sets, which were incorporated into least absolute selection and shrinkage operator (LASSO) regression for signature establishment. Following the grouping of LIHC samples according to risk score, the correlations between the signature and clinicopathological, tumour immune infiltration, and mutational characteristics as well as therapeutic response were also analysed. lncRNA expression levels used for modelling were finally examined at the cellular and tissue levels by real-time PCR. All analyses were based on R software.

**Results:**

AL031985.3 and MKLN1-AS were ultimately identified as signature-related lncRNAs, and both were significantly upregulated in HCC tissue samples and cell lines. The prognostic value of the signature reflected by the AUC value was superior to that of age, sex, grade and stage. Correlation analysis results demonstrated that high-risk groups exhibited significant enrichment of immune cells (DCs, macrophages and Tregs) and increased expression levels of all immune checkpoint genes. Prominent differences in clinicopathological profiles, immune functions, tumour mutation burden (TMB) and drug sensitivity were noted between the two risk groups.

**Conclusions:**

Our signature represents a valuable predictive tool in the prognostic management of HCC patients. Further validation of the mechanisms involved is needed.

**Supplementary Information:**

The online version contains supplementary material available at 10.1186/s12885-022-10049-w.

## Introduction

HCC is one of the subtypes of liver cancer, accounting for 90% of all cases [1, 2]. HCC has typically been associated with high mortality over the past decades due to the lack of effective approaches for early diagnosis and treatment. Currently, HCC is the sixth most common cancer worldwide, and its incidence and mortality continue to increase [3]. Generally recognized risk factors attributed to the occurrence and progression of HCC include long-term alcohol consumption, nonalcoholic steatohepatitis, and hepatitis virus infection [4]. The prolonged presence of these factors makes individuals at high risk of developing cirrhosis, a state that predisposes them to progress to advanced liver cancer with a poor prognosis. Therefore, the earlier the biomarkers associated with cancer progression are identified, the more likely it is that early intervention in HCC will be possible.

The ECM is a complex and dynamic scaffold network of secreted macromolecular substances. In addition to supporting and connecting tissue structures, the ECM regulates many crucial cellular physiological activities, including growth, migration, differentiation and apoptosis [5]. Evidence supports the overview that alterations in the structure and composition of the ECM allow cancer cells to achieve numerous hallmarks of cancer [6]. Additionally, by interfering with the drug transport and delivery process, the ECM may be involved in drug resistance mechanisms to some degree [7]. Based on improvements in sequencing technology, an increasing number of lncRNAs that may affect gene expression at the transcriptional and posttranscriptional levels have been discovered. The pivotal roles that lncRNAs may play in maintaining the cellular microenvironment and in the interaction between malignant cells and other cells are also widely understood [8]. This interaction involves alterations in fibril production to regulate fibroblast viability and apoptosis [9–11]. Given their critical role in the ECM, lncRNAs have been identified as key targets for HCC research. Therefore, in this study, we developed a prediction model based on ECM-related lncRNAs to analyse sample information downloaded from TCGA database with the aim of predicting the probability of survival in HCC patients.

## Materials and methods

### Data download, preliminary processing, and screening of ECM-related LncRNAs (ECMrlncRNAs)

We downloaded RNA sequencing (RNA-seq) and clinical data of liver cancer from The Cancer Genome Atlas (TCGA)-LIHC dataset (https://gdc.cancer.gov/). Then, a systematic collation was performed for subsequent analysis. LncRNA sets were extracted in bulk from the transcriptome profiles using GTF files and then assembled into an expression matrix. GTF files were obtained from the Ensembl website (http://asia.ensembl.org) for annotation to distinguish lncRNAs from the RNA-seq data. A list of ECM-related genes was retrieved from the Matrisome Project (www.matrisomeproject.mit.edu/other-resources/human-matrisome). Coexpression analysis was subsequently applied to screen ECM-related lncRNAs (ECMrlncRNAs). Those with correlation coefficients greater than 0.4 and *p* values less than 0.001 were ultimately identified as ECMrlncRNAs. Corresponding calculations were primarily based on the “limma” package in R.

### Construction of a prognostic signature using the LASSO algorithm

Hazard ratios (HR) of ECMrlncRNAs were calculated using univariate Cox hazard regression analysis. ECMrlncRNAs were considered eligible and incorporated into the LASSO algorithm for modelling if they were determined to have prognostic value based on HR values. LASSO Cox regression involves a penalized linear model with a shrinkage penalty that induces sparsity of predictors in the model. After completing the cross-validation, the LASSO results were optimized by multivariate regression analysis and then integrated into the model construction. Notably, the results of the cross-validation represent the number of lncRNAs that are deemed suitable for modelling by LASSO regression. The following formula was used for modelling: ${\sum}_{i=1}^n\left(\ expression\ level\ of\ lncRNA\ast regression\ coefficient\right)$. The R packages “survival” and “glmnet” were utilized for all calculations. The 1-, 3-, and 5-year ROC curves were subsequently plotted to show the prognostic performance of the signatures using the “survival”, “survminer” and “timeROC” packages. When the model was determined to exhibit favourable prognostic value, total samples included in the analysis were divided into training and testing groups for the subsequent analyses. Each group was divided into a high-risk set and a low-risk set based on the median risk score. To further illustrate the distribution of the two risk groups, principal component analysis (PCA) was applied for visualization. In addition, Kaplan–Meier analysis was also performed with the “survival” package to reflect the difference in overall survival (OS). The relationship between the signature and clinicopathological characteristics was assessed primarily based on chi-square tests. A combined analysis of univariate regression and multivariate regression was conducted to determine the independent risk factors.

### Functional enrichment exploration

To assess the contribution of differentially expressed genes (DEGs) to a phenotype, we adopted the “clusterProfiler” package for GO term and KEGG pathway analyses [12–14]. The thresholds were set as a *P* value < 0.05 and FDR < 0.05. The results are presented as bar plots.

### Immune correlation analysis

Using the median risk score as a cut-off, we divided the HCC patients into two subgroups: high- and low-risk groups. The relationships between the established signature and tumour immune features were analysed by ssGSEA and TIMER. The use of these algorithms in this procedure helped to reveal differences in the distribution of tumour-infiltrating immune cells and immune-related functions between the two risk groups. Moreover, we performed a correlation analysis of the risk score with cytokines and chemokines based on the gene expression level. These included cytokines and chemokines were obtained from Ozga et al. and Montanari et al. [15, 16]. A *P* value< 0.05 was considered a statistically significant threshold.

### The relationship between the signature and TMB

TMB is defined as the total number of substitutions and insertions/deletions per megabase (Mut/Mb) in the exon coding region of the evaluated gene in one tumour sample. TMB is implicated in overall survival after immunotherapy in multiple cancer types, suggesting the importance of TMB as a predictive biomarker for the efficacy of immune checkpoint inhibitors (ICIs) [17]. To examine the predictive value of TMB in our model, we obtained masked somatic mutation (MSM) data from the TCGA-SNP category. After processing the data analytically, the R package “maftools” was used to analyse and visualize the mutation frequencies and types of genes between both risk groups.

### The association between the signature and immune checkpoint genes

Linking the signature to the expression level of various immune checkpoint genes contributed to the identification of appropriate therapeutic options for HCC populations at different levels of risk. The Wilcoxon signed-rank test was applied in the comparison of gene expression levels in different risk groups using the R package “ggstatsplot”, and the corresponding results were presented as box plots.

### Prediction of therapeutic response

Comparison of drug sensitivity to targeted drugs and chemotherapeutic agents in the high- and low-risk groups was accomplished by calculating IC_50_ values using the R package “pRRophetic”. The drugs included in the comparison are routinely applied in the treatment of HCC, such as sorafenib, sunitinib, erlotinib, bleomycin, cisplatin, doxorubicin, gemcitabine, mitomycin C, docetaxel, paclitaxel, temsirolimus and vinblastine. To establish a link between our signature and the immunotherapeutic response, we used the Tumour Immune Dysfunction and Exclusion Algorithm (TIDE) to model and integrate two key mechanisms of tumour immune evasion to provide predictive results for immunotherapy. Elevated TIDE predicts that patients have suppressive cells that inhibit T-cell infiltration and fail to respond to immunotherapy [18].

### Cell culture and real-time PCR

The normal cell line (L02) and HCC cell lines (SK-Hep-1, Hep-G2, Huh-7 and LM-3) were purchased from the Key Laboratory of Biotherapy and Regenerative Medicine, Gansu Province (Gansu, China). All cell lines were cultured in Dulbecco’s modified Eagle’s medium (DMEM, Solarbio, Beijing, China) with 10% foetal bovine serum (FBS, Solarbio) and cyan chain double antibodies (Beyotime, Jiangsu, China) in a 37 °C humidified atmosphere that contained 5% CO_2_. When the HCC cell lines achieved the indicated conditions, total RNA extraction was performed with TransZol (TransGen, Beijing, China) following the manufacturer’s instructions. The isolation and purification of cytoplasmic and nuclear RNA was principally accomplished using an EasyPure RNA kit (TransGen). The concentration of purified RNA was then determined using a NanoDrop 2000 spectrophotometer (Thermo Fisher Scientific, MA, USA). To achieve cDNA synthesis, we employed a TransScript One-Step gDNA Removal and cDNA Synthesis SuperMix (TransGen) to reverse transcribe RNA that was previously extracted. HCC and adjacent tissue cDNA microarrays were obtained from Shanghai Outdo Biotech Company, China. Finally, real-time PCR was performed in TransStart Top Green qPCR SuperMix (TransGen). Quantification of the RNA expression level was realized using the 2-ΔCt method (ΔCt = Ct (Target) - Ct (Reference)). The primer sequences are as follows: AL031985.3 forward primer AAATCCCATACCCCTTTCACC reverse primer TTTACTGAGTCCCTTCTGCGTG; MKLN1-AS forward primer GTGTTTCTCTCTGAAAGCAGCG, reverse primer TTCAAAAGTGACCAAAGCCAGG; GAPDH forward primer GTCAAGGCTGAGAACGGGAA, reverse primer AAATGAGCCCCAGCCTTCTC; β-actin (validated and provided by Outdo Biotech) forward primer GAAGAGCTACGAGCTGCCTGA, reverse primer CAGACAGCACTGTGTTGGCG. The ethics statement for this study was provided by Shanghai Otto Biotech.

### Statistical analysis

All analyses were implemented based on R software (version 3.6.3). A *P* value < 0.05 was considered statistically significant.

## Results

### ECMrlncRNA screening and prognostic signature construction

**Fig. 1:**
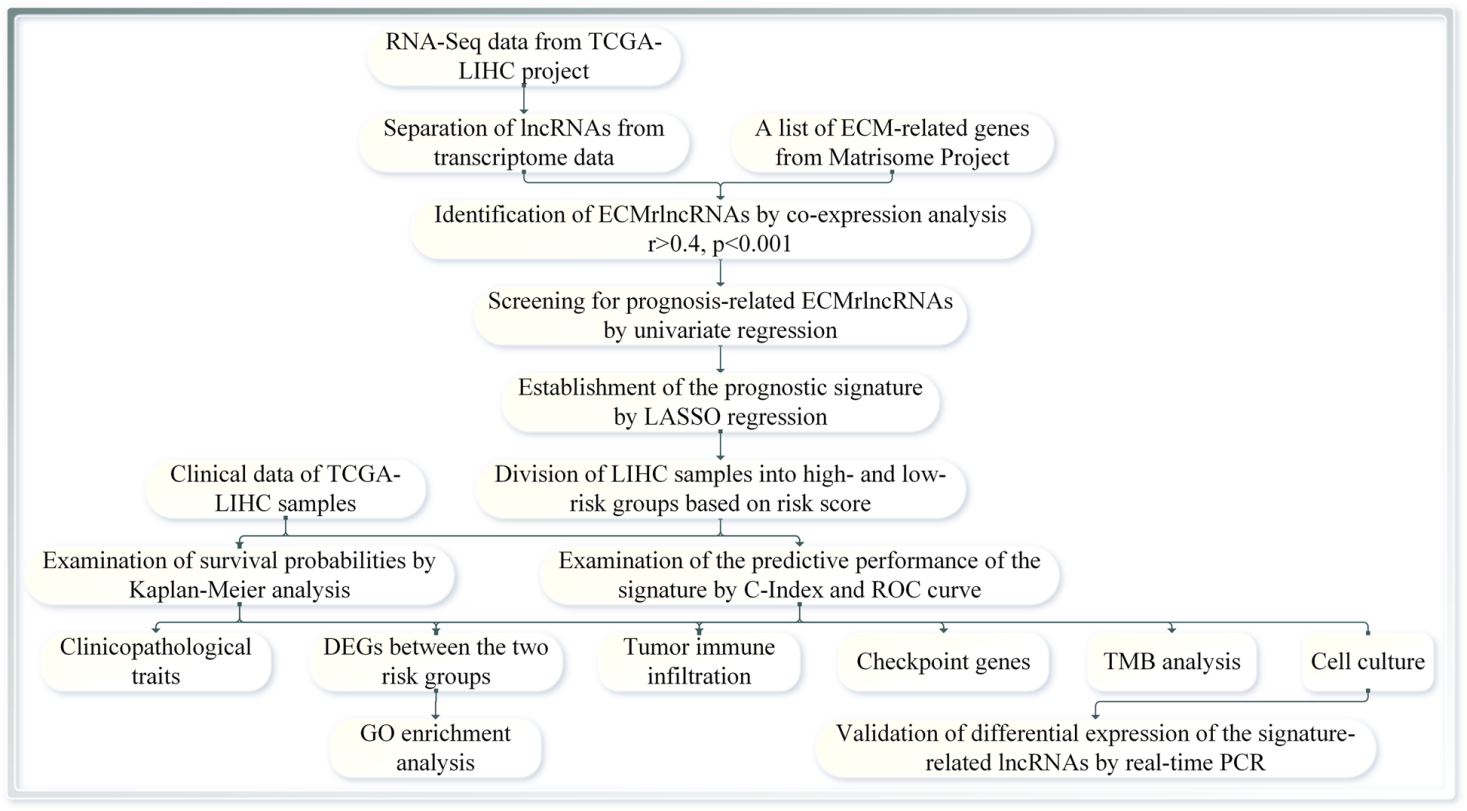
Study Workflow

**Fig. 2:**
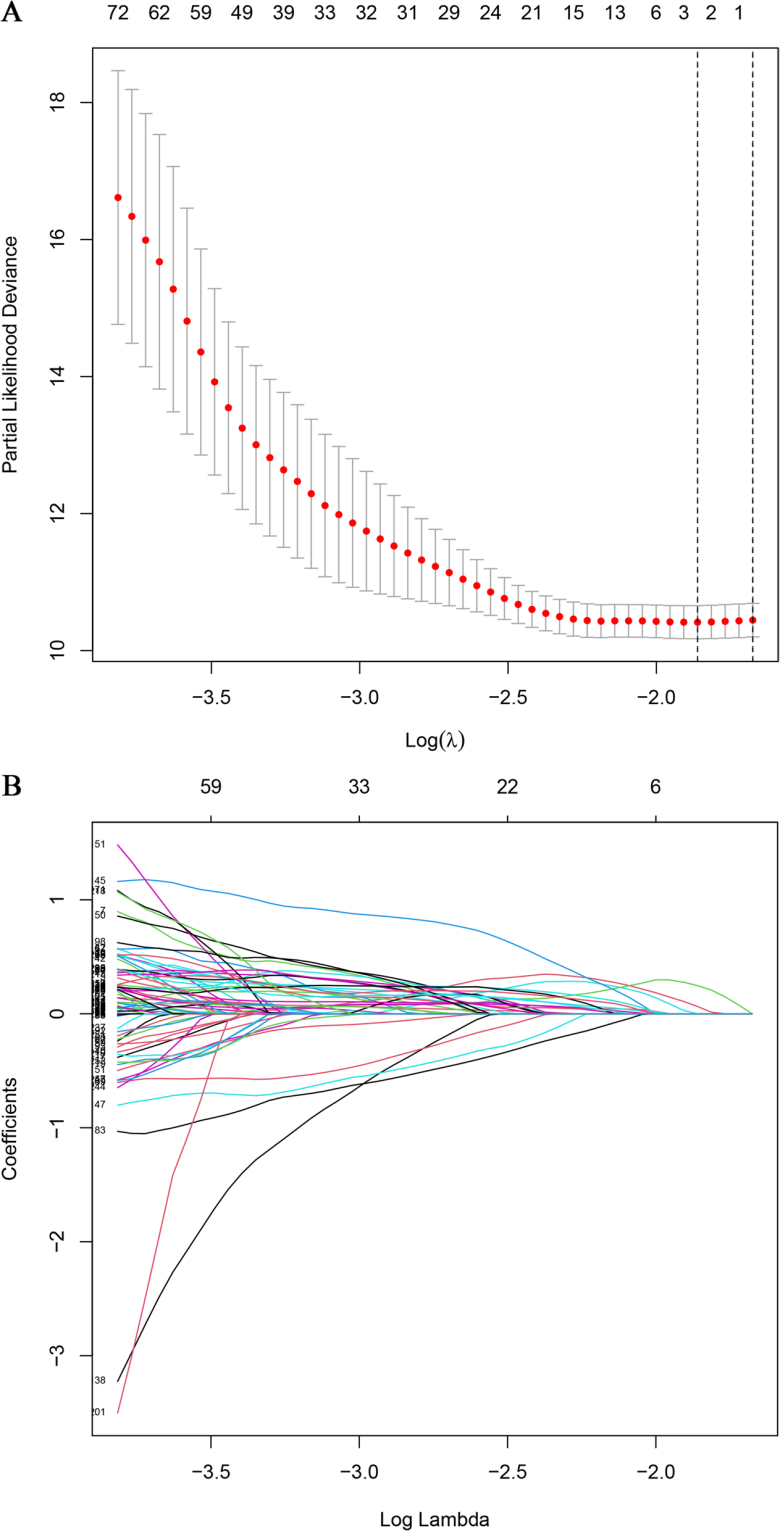
Cross-validation and LASSO Regression. When the curve reaches the lowest point, the error of cross-validation is minimized. At this point, the corresponding figure represents the number of significant lncRNAs (**A**). LASSO regression plot (**B**)

**Fig. 3:**
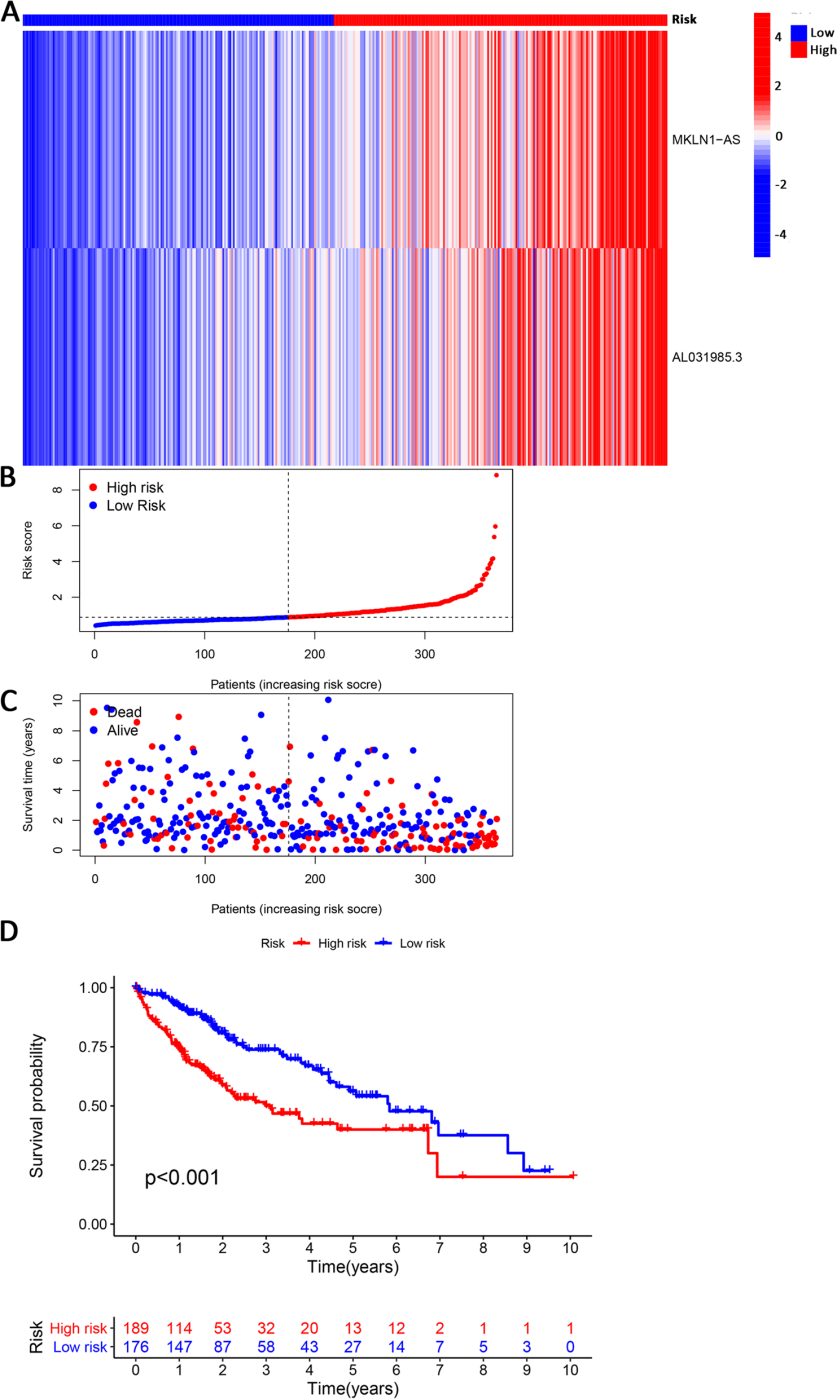
Identification of Prognosis-Related ECMrlncRNAs and Signature Development. Heatmap visualizing the expression levels of MKLN1-AS and AL031985.3 in the total LIHC cohort (**A**). PCA displays the distribution of different-risk patients and differences in survival status (alive or dead) (**B** and **C**). Survival curves revealed the prognostic differences between the high- and low-risk HCC groups (**D**)

The study scheme is presented in Fig. 1. We initially obtained a total of 1068 ECM-related gene sets from the Matrisome Project. Following further coexpression analysis between the ECM-related genes and lncRNA sets, 1961 lncRNAs were eventually identified as ECMrlncRNAs. Through subsequent univariate Cox regression, lncRNAs with prognostic value were formally included in the LASSO regression analysis (cross-validation and regression plots are shown in Fig. 2A and B, respectively). Two ECMrlncRNAs (MKLN1-AS and AL031985.3) that demonstrated distinct expression differences between tumour and normal tissues were included in the risk model. The model formula is described as follows: risk score = 0.942362440899263* mRNA expression level of MKLN1-AS+ 0.756996665090891* mRNA expression level of AL031985.3. LIHC samples were divided into training and testing groups in our analysis. However, comparative analysis concerning the baseline characteristics of the three groups revealed no significant differences (Table 1). Figure 3A represents the expression levels of two lncRNAs in the total sample group. Dimensionality degradation via PCA allowed for a clearer view of the distribution of patients from the total LIHC sample with different risk scores (Fig. 3B) and the corresponding differences in patient survival status (alive or dead) (Fig. 3C). Immediately afterwards, we performed Kaplan–Meier analysis to assess the survival differences, the results of which are presented as survival curves (Fig. 3D). The findings indicated that patients with higher risk scores have lower OS (*P* < 0.001). Similar lncRNA expression (Fig. S1A and E), patient distribution (Fig. S1B and F), patient survival status (Fig. S1C and G) and OS (Fig. S1D and H) results were observed in the total LIHC group as well as the training and testing groups.

**Table 1:** Demographic and baseline disease characteristics of samples in the TCGA-LIHC project

Covariates	Total	Training set	Testing set	*P* value*
Age - no. (%)				1
<=65	227(62.19%)	114(61.96%)	113(62.43%)	
> 65	138(37.81%)	70(38.04%)	68(37.57%)	
Gender - no. (%)				0.9091
Female	119(32.6%)	61(33.15%)	58(32.04%)	
Male	246(67.4%)	123(66.85%)	123(67.96%)	
Grade - no. (%)				0.8485
G1	55(15.07%)	27(14.67%)	28(15.47%)	
G2	175(47.95%)	92(50%)	83(45.86%)	
G3	118(32.33%)	58(31.52%)	60(33.15%)	
G4	12(3.29%)	5(2.72%)	7(3.87%)	
Unknow	5(1.37%)	2(1.09%)	3(1.66%)	
Stage - no. (%)				0.163
Stage I	170(46.58%)	79(42.93%)	91(50.28%)	
Stage II	84(23.01%)	41(22.28%)	43(23.76%)	
Stage III	83(22.74%)	44(23.91%)	39(21.55%)	
Stage IV	4(1.1%)	4(2.17%)	0(0%)	
Unknow	24(6.58%)	16(8.7%)	8(4.42%)	

*P value less than 0.05 is considered to be statistically significant

### Assessment of the predictive value of the signature

**Fig. 4:**
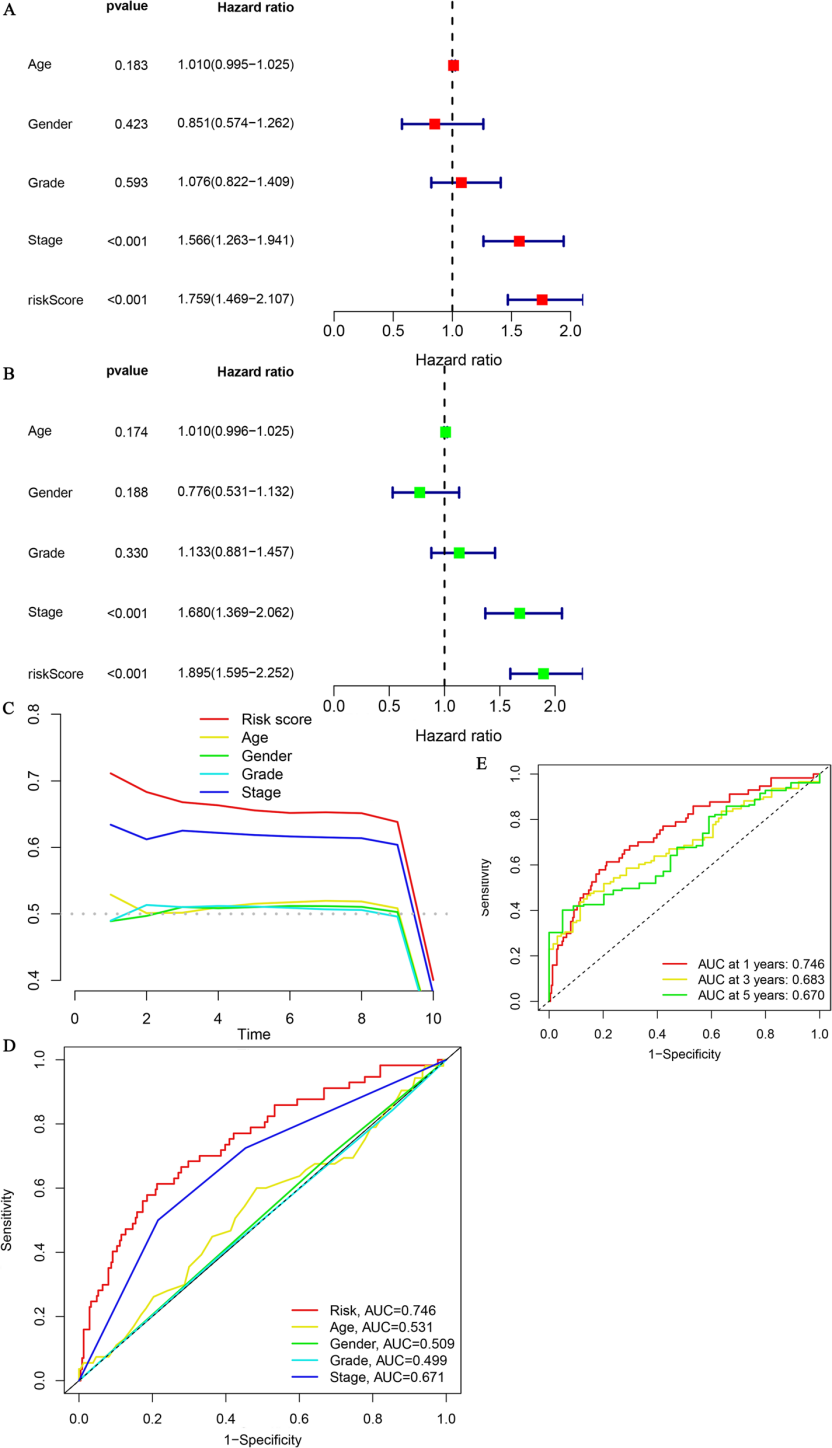
Examination of the Predictive Properties of the Signature. Univariate (**A**) and multivariate (**B**) regression analyses indicated that the stage and risk score were independent risk factors. The predictive value of the signature was illustrated by the concordance index (**C**) and ROC curves. ROC curves comparing the signature with age, sex, grade and stage showed the superiority of the risk score compared with other indicators (**D**). ROC curves at 1, 3 and 5 years were also plotted to reflect the long-term predictive value (**E**)

The rationale for signature construction based on the risk score was derived mainly from the results of univariate and multivariate regression analyses, which suggested that the risk score could serve as an independent risk factor for liver cancer (Fig. 4A and B). To further illustrate the use of the risk score as an important reference for model construction, we compared it with commonly available clinical predictors. The risk score showed a comparatively superior diagnostic ability compared with age, sex, grade and stage (Fig. 4C and D). The AUC values representing the prognostic predictions at 1, 3 and 5 years were 0.746, 0.683 and 0.670, respectively (Fig. 4E).

### Application of the signature to TCGA-LIHC datasets

**Fig. 5:**
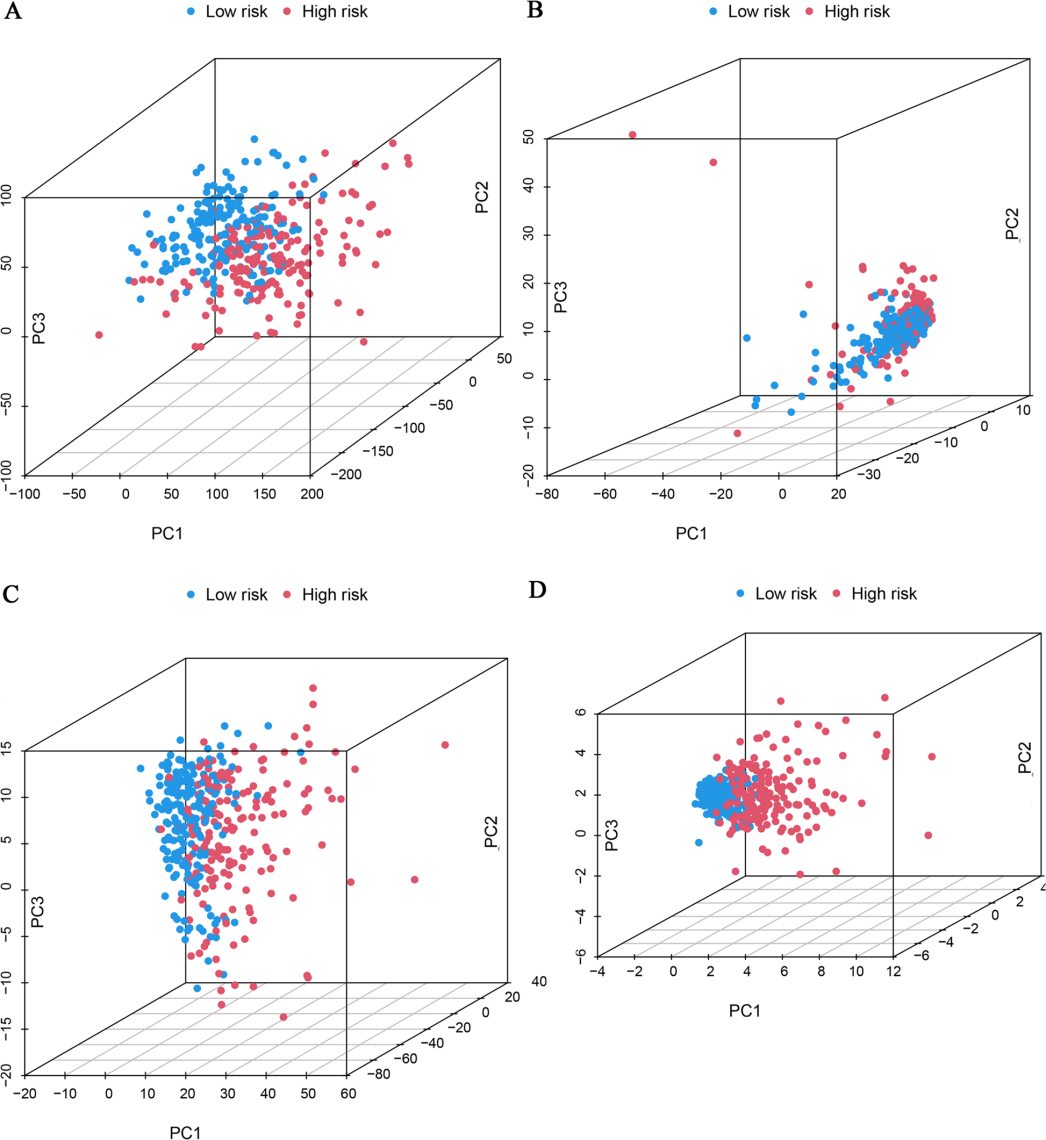
PCA Analysis for Visualization of Distribution Difference. PCA demonstrated that compared to all genes (**A**), ECM genes (**B**) and ECMrlncRNAs (**C**), the risk score (**D**) was able to significantly distinguish between high-risk and low-risk patients

The constructed model employed a risk score-based cut-off criterion, namely, the median risk score, to divide HCC samples into high- and low-risk groups. Analysis of the relationship between the signature and clinicopathological profiles illustrated significant differences between the two risk groups in terms of age, sex, grade and TNM stage (Fig. S2A-O). Subsequently, we employed the PCA approach to confirm the strength of the risk score in separating patients with different risks, demonstrating the superiority of the risk score in accurately distinguishing samples compared with other indicators (all genes, ECM-related genes and ECMrlncRNAs) (Fig. 5A-D).

### Functional enrichment analysis

**Fig. 6:**
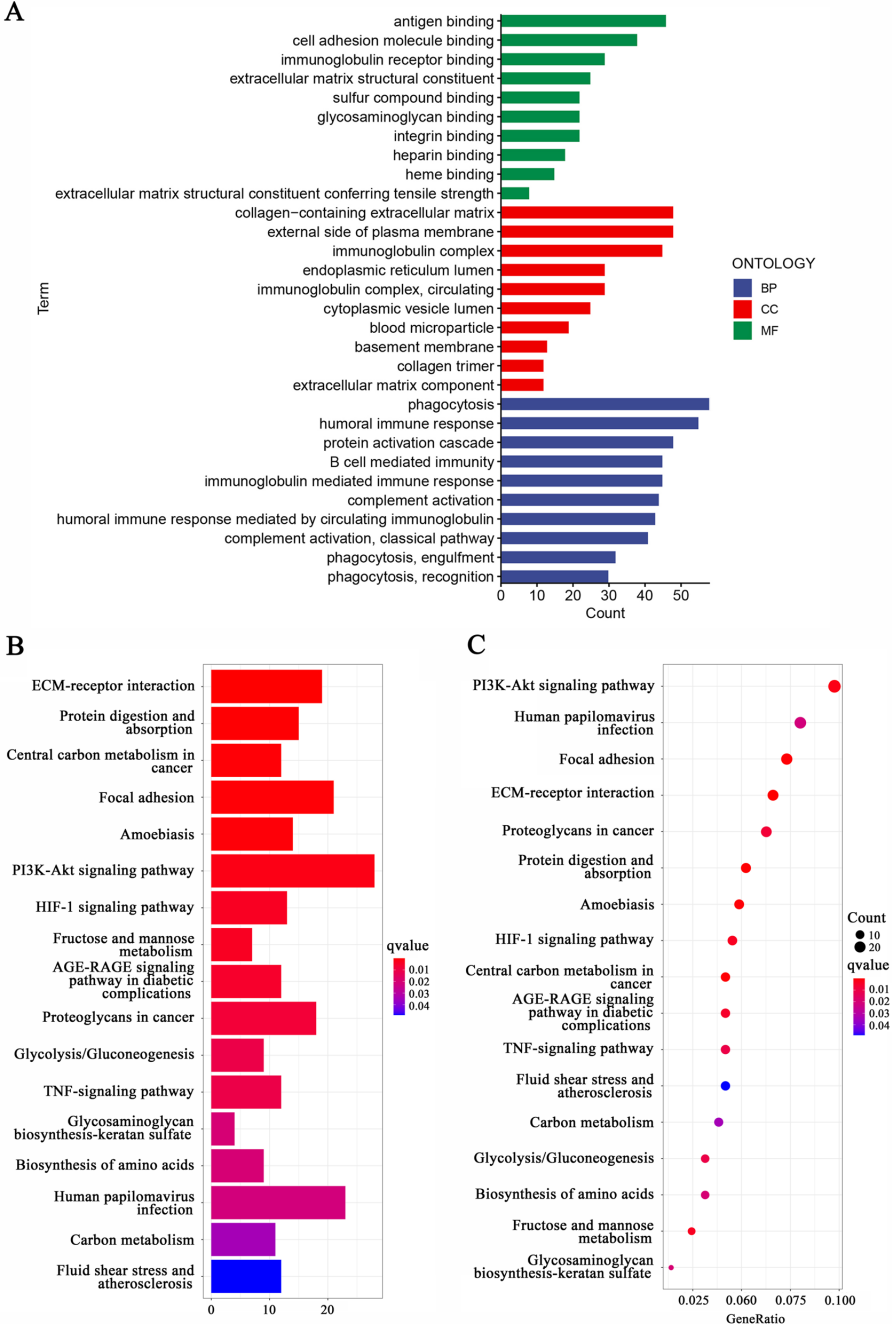
Go and KEGG Enrichment Analysis. The GO terms are classified into three categories, BP, CC and MF, as shown in Panel (**A**). KEGG pathway analysis results are presented as a bar plot (**B**) and bubble plot (**C**). The horizontal coordinate represents the number of genes enriched in each GO term/KEGG pathway. The vertical coordinate denotes the full name of each GO term/KEGG pathway

The biological functions of the 636 DEGs identified between the two risk groups were assessed. GO terms are classified into three subtypes: BP, CC and MF. BP primarily included antigen binding, cell adhesion molecule binding, immunoglobulin receptor binding, and extracellular matrix structural constituent. CC mainly included collagen-containing extracellular matrix, the external side of the plasma membrane, and immunoglobulin complexes. MF comprised phagocytosis, humorall immune response, and protein activation cascade (Fig. 6A). Using KEGG analysis, 17 pathways were ultimately identified, including ECM-receptor interaction, focal adhesion, and PI3K-Akt signalling pathway (Fig. 6B and C).

### Relationship between the signature and tumour immune-infiltrating features

**Fig. 7:**
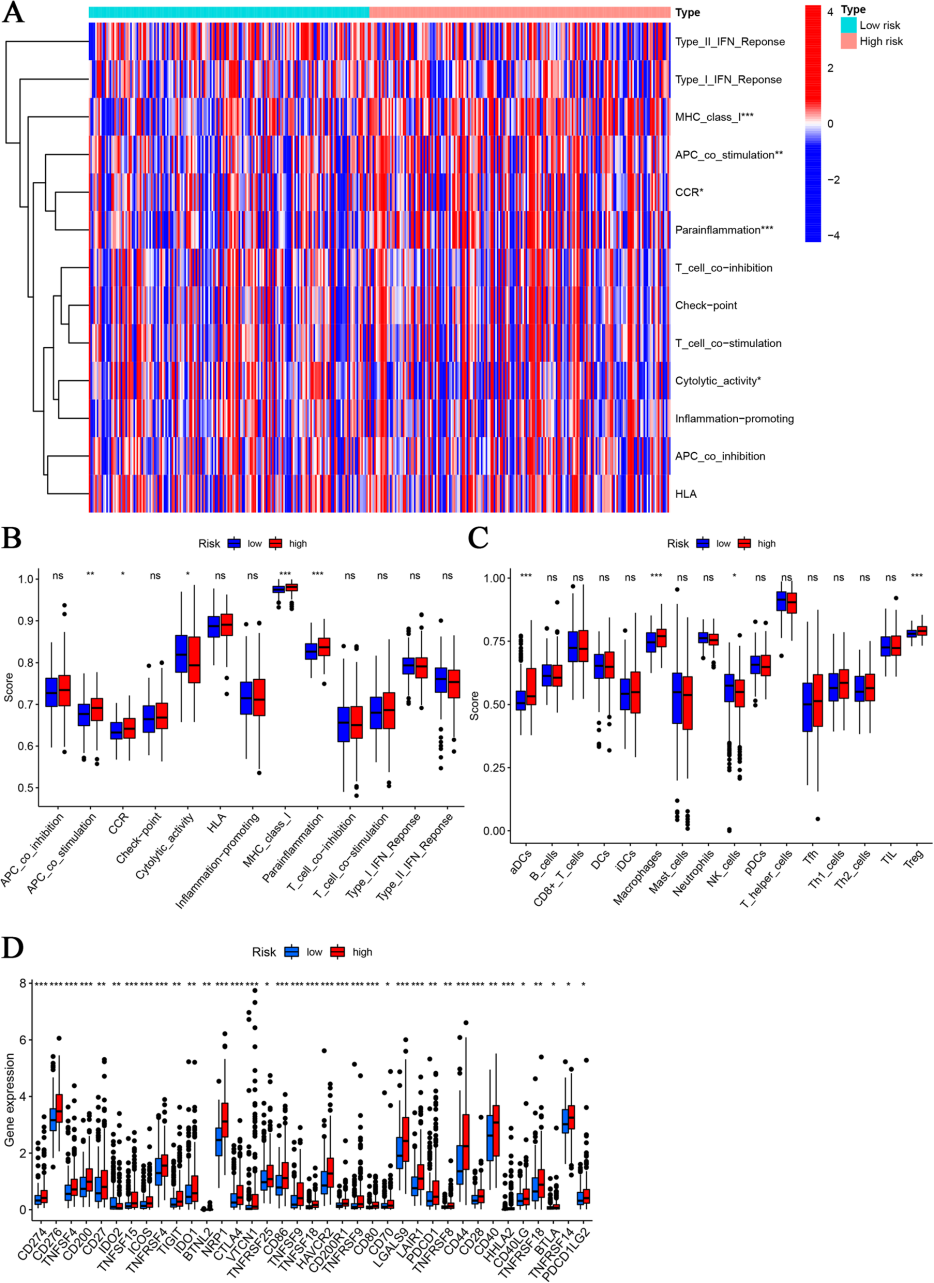
Differential Analysis of Immune function, Tumour Infiltrating Immune Cells and Immune Checkpoint Genes. The distinctions in immune-related functions between the high- and low-risk groups are presented as a heatmap (**A**) and box plots (**B**). The differences in tumour-infiltrating immune cells between the two groups are reported as box plots using the ssGSEA algorithm (**C**). Differences in the expression of immune checkpoint genes in the two groups are presented as box plots (**D**). “*”, “**” and “***” represent *P* < 0.05, *P* < 0.01 and *P* < 0.001, respectively

Detailed differences in immune-related functions, immune cell subtypes and checkpoint genes were analysed between the two groups. The results suggested that MHC class I, APC costimulation, CCR, parainflammation and cytolytic activity were more significantly correlated with high risk scores (Fig. 7A and B). In contrast to NK cells, DCs, macrophages and Treg cells were prominently enriched in the high-risk group (Fig. 7C). The TIMER algorithm also yielded similar results (Fig. S3A). Furthermore, all of the checkpoint genes of interest showed considerable overexpression in the high-risk group (Fig. 7D). Correlation analysis of the risk score with cytokines and chemokines revealed that the risk score was positively correlated with CXCL1, CXCL3, CXCL5, CXCL8, CXCL9, CXCL11, CCL2, CCL4, CCL20, CCL26, CCL28, IL6, IL10, TGFB2, FGF2, VEGFA and HGF and negatively correlated with CCL14 and CCL15 (Fig. S3B).

### Correlation of the signature and TMB

**Fig. 8:**
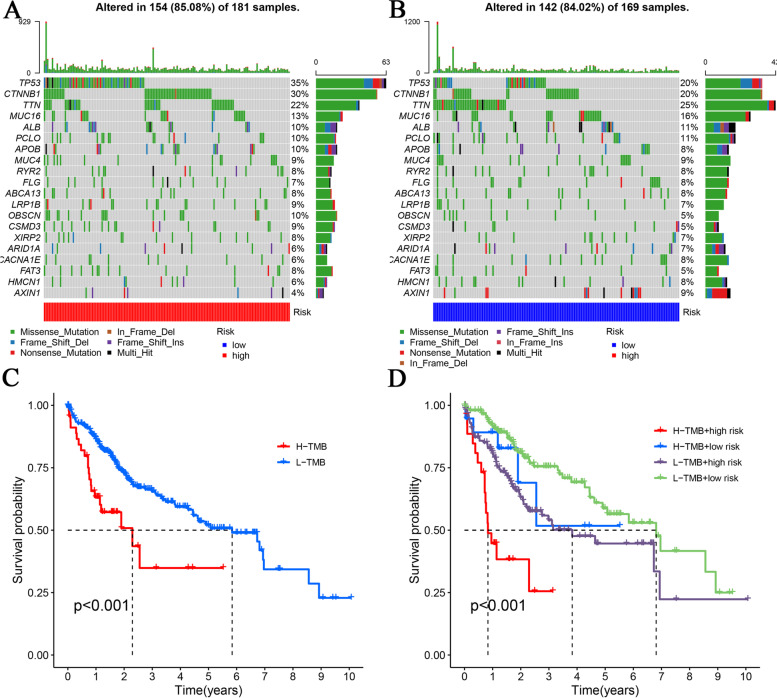
Comparison of Gene Mutation Frequencies and Survival Status. Waterfall plots were used to show the mutation frequencies of genes in the high-risk (**A**) and low-risk (**B**) groups. High risk is indicated in blue, and low risk is indicated in red. Comparison of survival probabilities between groups clustered according to TMB (**C**) or TMB combined with risk score (**D**)

Given the crucial role that TMB plays in cancer progression, we report the mutation frequencies of the top 20 driver genes in the high- and low-risk groups, separately (Fig. 8A and B). Accordingly, the low-TMB group had a comparatively higher survival probability when grouped exclusively by TMB, whereas the low-TMB and low-risk group had a superior prognosis when grouped according to a criterion based on TMB in combination with the risk score (Fig. 8C and D).

### Drug sensitivity and immune response prediction

**Fig. 9:**
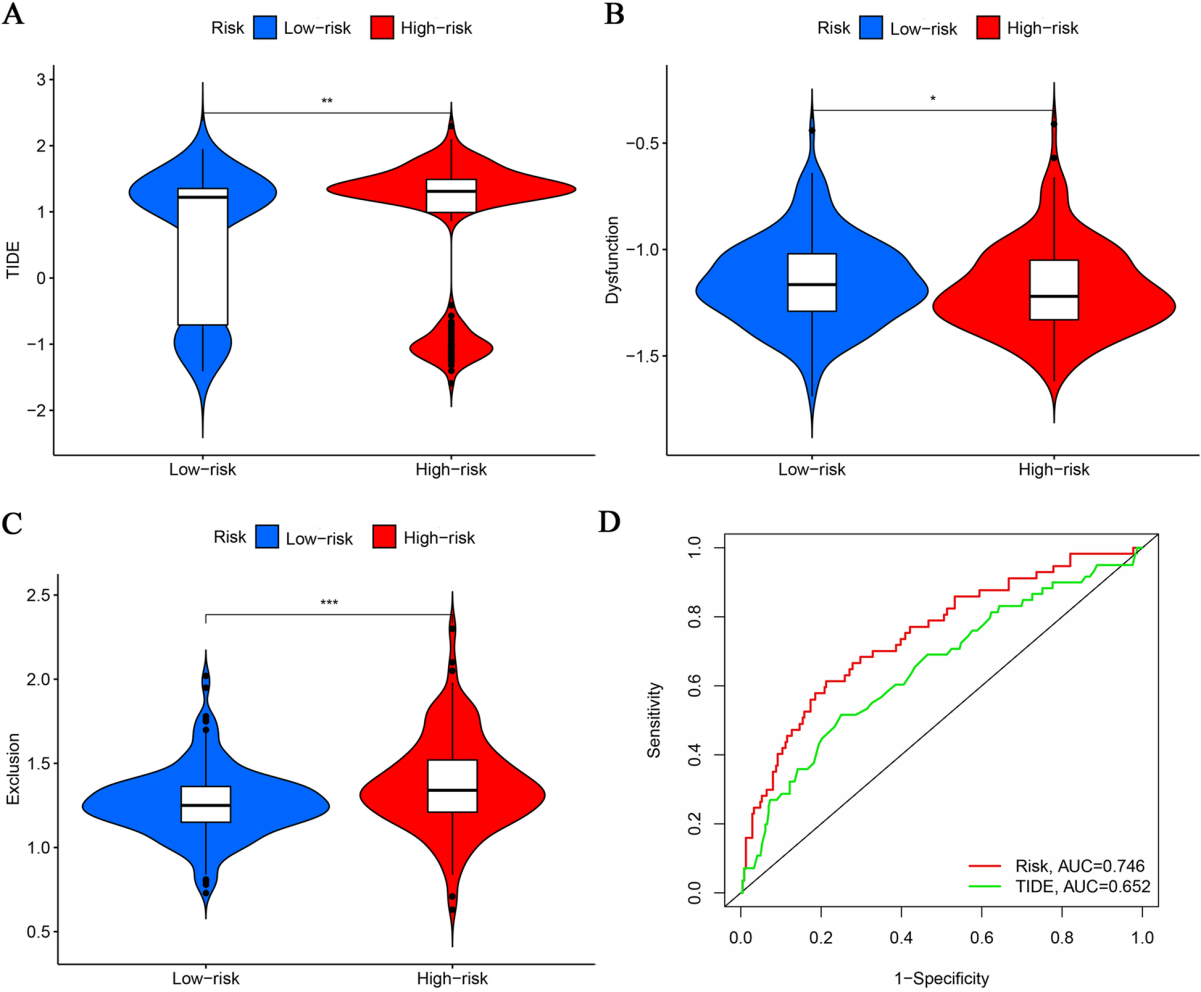
Immunotherapy Response Prediction and Signature Comparison. The comparisons between the two risk groups in terms of TIDE score (**A**), Dysfunction score (**B**) and Exclusion score (**C**) are presented as violin plots. ROC is plotted to compare the predictive value of the risk score and the TIDE score (**D**)

Sensitivity analysis of several chemotherapeutic and targeted therapeutic agents, including bleomycin, cisplatin, doxorubicin, and mitomycin C, was performed. Patients in the high-risk group were more sensitive to paclitaxel and gemcitabine based on lower IC_50_ values, whereas patients in the low-risk group were sensitive to sorafenib, docetaxel, erlotinib, and temsirolimus based on higher IC_50_ values. No significant differences in sensitivity to sunitinib and vincristine were observed between the two groups (Fig. S4A-L). To compare the difference in response to immunotherapy between the high-risk and low-risk groups, we then performed an analysis by employing the TIDE algorithm (Fig. 9A). The TIDE score is composed of two components: a dysfunction score and exclusion score. Here, the high-risk group exhibits a higher dysfunction score (Fig. 9B), and the low-risk group exhibits a higher exclusion score (Fig. 9C). Overall, the high-risk group had a higher TIDE score (Fig. 9A), implying that it may be associated with poorer immune checkpoint inhibition therapeutic efficacy. Next, we compared the predictive value of our model with the TIDE score, and the results are shown in Fig. 9D.

### Exploration and verification of signature-related LncRNA expression profiles

**Fig. 10:**
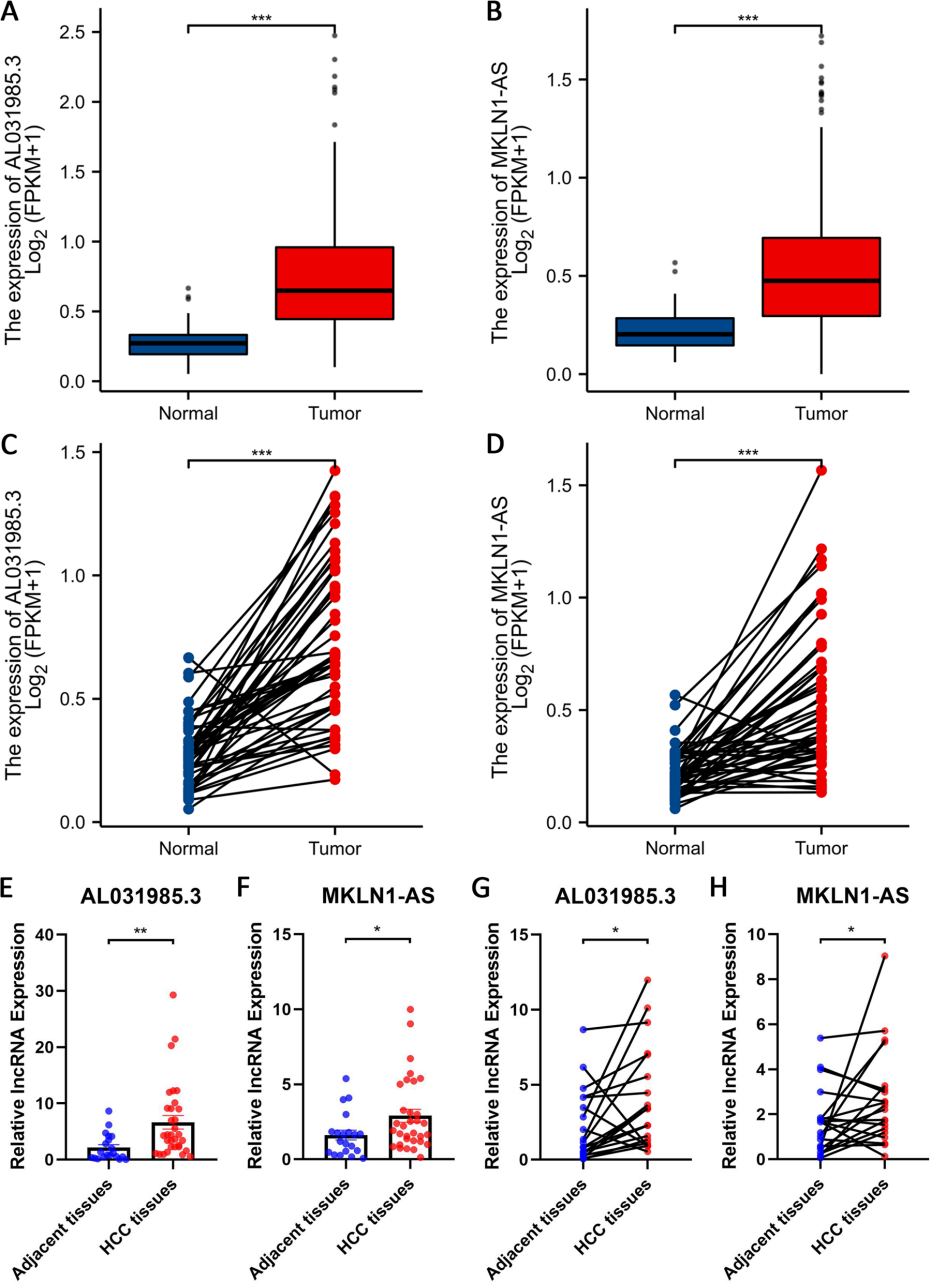
Assessment of Differential Expression of LncRNAs Used for Modelling. Comparison of the differential expression of AL031985.3 (**A**) and MKLN1-AS (**B**) between tumour and normal samples from the TCGA-LIHC project. Paired comparison of the differential expression of AL031985.3 (**C**) and MKLN1-AS (**D**) between tumour and normal samples from TCGA-LIHC project. Unpaired comparison of the differential expression of AL031985.3 (**E**) and MKLN1-AS (**F**) between 32 HCC tissues and 20 adjacent tissues. Paired comparison of the differential expression of AL031985.3 (**G**) and MKLN1-AS (**H**) between 20 HCC tissues and 20 adjacent tissues

**Fig. 11:**
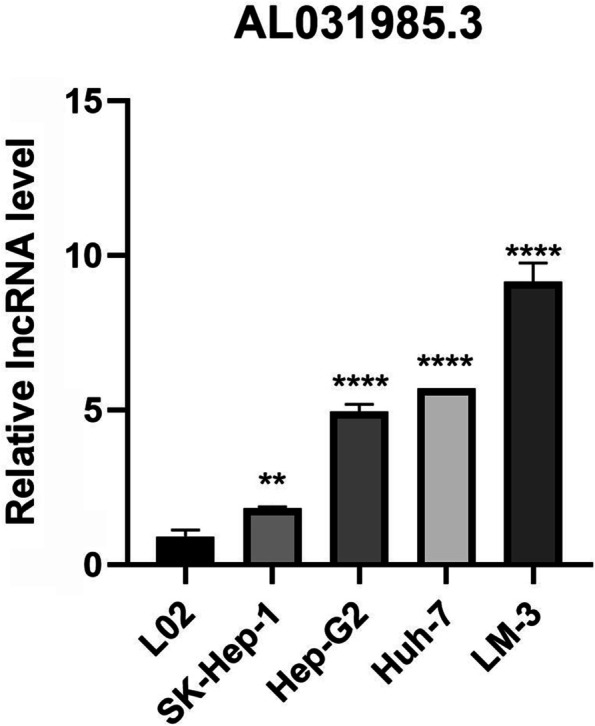
Assessment of Relative AL031985.3 Expression Levels in Various Cell Lines via Real-time PCR. L02 is a normal cell line. SK-Hep-1, Hep-G2 and Huh-7 are well differentiated, whereas LM3 is poorly differentiated. “**” and “****” represent *P* < 0.01 and *P* < 0.0001, respectively

Both overall and pairwise comparisons demonstrated significantly higher expression levels of AL031985.3 in the tumour group than in the normal group (Fig. 10A and C). Validation results at the cellular level also provided support for this finding (Fig. 11). In addition, based on our bioinformatics analysis, MKLN1-AS was also predicted to be overexpressed in tumour tissues (Fig. 10B and D). Verification results have been presented in a previous study. To confirm the aforementioned findings, 20 pairs of HCC samples (containing 20 tumour tissues and 32 adjacent tissues) were assembled and utilized for quantitative analysis of lncRNAs. Validation of these samples showed that AL031985.3 (Fig. 10E and G) and MKLN1-AS (Fig. 10F and H) were significantly upregulated in HCC in both pairwise and unpaired analyses.

## Discussion

Given the pivotal role that ECM components play in tumour onset and progression, a comprehensive understanding of their biophysical and biochemical effects and remodelling processes seems particularly imperative to uncover promising biomarkers for diagnostic and therapeutic applications. Database mining using various algorithms and accurate prediction models in the context of big data are of great help in the discovery of lncRNAs that are relevant to ECM. Therefore, this study identified ECMrlncRNAs to provide a prognostic prediction approach for various risk populations to rationally guide clinical decision-making.

Despite the existence of several valuable signatures for the prognostic analysis of HCC patients, [19–28], our study differs from previous studies based on various features, such as the prognostic value of the signature, the ability to accurately differentiate samples and the correlation with immune landscape. The principal cause of this difference was based on our selection of a lncRNA set that was coexpressed with ECM-related genes. Two signatures consisting of ECM-related genes have been previously reported [24, 25]; however, these signatures were not equivalent to our lncRNA signature. Indeed, comprehensive studies were performed to evaluate the prognostic value of the models generated using these signatures, and these signatures also predicted the immune landscape as well as drug sensitivity. In contrast to the study by Tang et al., our study focused on assessing the differences in drug sensitivity to sorafenib in the risk subgroup obtained from LIHC samples. Given that sorafenib is currently the first-line drug for HCC treatment, it would be more clinically useful to construct a model that demonstrates a better association with this drug. More importantly, following a series of screening processes according to the developed criteria, two lncRNAs, AL031985.3 and MKLN1-AS, were eventually identified and incorporated into the signature. We subsequently validated these two lncRNAs at the cellular and tissue levels and further examined their prognostic value in HCC. Thus, our findings are more reliable and valuable. The upregulation of AL031985.3 and MKLN1-AS in HCC was identified through database mining but was also confirmed in a series of in vitro experiments. Few experimental studies have been performed on AL031985.3, and its function in HCC requires further exploration. Comparatively, several recent studies demonstrated phenotypic alterations mediated by MKLN1-AS. MKLN1-AS is potentially involved in the regulation of cell proliferation, angiogenesis, migration and invasion [26–28] and implicated in poor survival [28]. Mechanistically, MKLN1-AS may function as a competing endogenous RNA to induce pro-oncogenic effects during HCC progression [28]. Overall, the signature had favourable prognostic performance, as evidenced by the ROC and corresponding AUC values at 1, 3 and 5 years. Accordingly, we divided HCC populations into high- and low-risk groups based on the risk score cut-off value. Our signature was also applicable to HCC patients with specific clinicopathological characteristics. Patients with lower risk scores generally had a better prognosis, regardless of stratification by age, sex, grade or TNM stage. In addition, the DEGs identified between the two risk groups were then entered into the GO analysis, and these genes had robust associations with ECM and immune functions.

Considering the poorly characterized immune microenvironment in HCC [29], the risk score determined in this study is a valuable marker in the differentiation of populations, particularly with respect to tumour-infiltrating immune cells, immune-related functions and immune checkpoint genes. We noted that the high-risk group was generally accompanied by a significant enrichment of immune cells associated with more immune functions and, more importantly, positively correlated with the expression level of immune checkpoint genes. Here, we identified DCs, Tregs, macrophages and CAFs as four hallmark immune cells given their remarkable abundance in the high-risk set. DCs in the tumour microenvironment (TME) may be associated with T-cell dysfunction through mediator release or checkpoint ligand expression, a mechanism by which tumours cooperate with their microenvironment to eclipse immune surveillance [30, 31]. Specifically, in HCC, Treg cells can be recruited into tumour tissues, and this accumulation is mainly mediated by the chemokine CCL22, which is secreted by intratumoural DCs [32]. Tumour-infiltrating DCs and Tregs promote immunotolerance and immune escape by suppressing effector T-cell responses, but high intratumoral numbers of these immune cells may be associated with poor prognosis [32, 33]. This notion is closely linked to our finding that increased levels of DCs and Tregs accumulated in the high-risk group (a high risk score was associated with a negative impact on clinical outcomes). Moreover, macrophages also showed increased enrichment in the high-risk group. The characterized enrichment of macrophages is notably linked to the survival inferiority of LIHC [30], indicating that this type of tumour-infiltrating macrophage can serve as a potential candidate cell for cancer therapies. Cancer-associated fibroblasts (CAFs) are also key players in the pathogenesis of HCC. In the HCC TME, CAF-derived CLCF1 enhances the secretion of the chemokines CXCL6 and TGF-β in HCC cells, which are subsequently capable of activating the ERK 1/2 signalling pathway in CAFs and stimulating the increased production of CLCF1. This positive feedback mechanism undoubtedly accelerates HCC progression and predicts a poor prognosis [34]. Additional findings also revealed a positive feedback loop between CAFs and the FOXQ1/NDRG1 axis that drives the initiation of HCC in tumour cells [35]. Furthermore, loss of CAF-derived exosomal miR-320a may partly contribute to HCC tumour proliferation and metastasis [36]. These observations objectively confirmed our assumption that high-risk HCC patients might have an advanced cancer stage with reduced survival probability. NK cells were present at significantly decreased levels in high-risk samples in our analysis, and these cells were also decreased in HCC tissues [37]. Of note, the number of intrahepatic NK cells is positively associated with poor clinical outcomes [38]. In addition to the abovementioned immune cells, differential expression of all included immune checkpoint genes was observed between the two groups. On the one hand, our results demonstrated that the accuracy and importance of patient clustering based on this criterion (risk score) is self-evident. In addition, the differential expression levels of these genes in the two risk sets revealed insight into the precise management of HCC. The most prominent immune checkpoint gene is cytotoxic T lymphocyte protein 4 (CTLA-4), which mediates immunosuppression and inhibits T-cell proliferation and the release of cytotoxic mediators [39–42]. Furthermore, it is also particularly important to perform correlation analysis between our models and chemokines and cytokines. Chemokines comprise a large family of at least 47 structurally related small cytokines, and their interaction with the extracellular matrix is specifically essential in controlling the directional migration of cells. In contrast, cytokines are key mediators of cellular communication in the TME and are closely associated with tumour development, progression and metastasis as well as the response to tumour therapy [42]. Overall, analysis of LIHC samples revealed underlying migration patterns of immune cells and regulatory mechanisms of immune surveillance. Given their crucial role in the regulation of immune responses, infiltrating immune cells, immune checkpoint genes, chemokines and cytokines represent attractive targets for tumour immunotherapy.

A critical and novel project introduced in this study was the evaluation of the correlation between TMB and the model. ICIs have revolutionized the field of cancer management by providing a new paradigm. Several attempts have been made to determine valuable predictive biomarkers, the most intriguing of which is TMB [17]. In this study, we examined the mutation frequencies of the top genes and focused on the survival probability of patients based on various groupings. Through our comparative analysis of TMB in different risk groups along with exploration of differences in the expression levels of immune checkpoint genes, precise and effective management of ICIs in HCC treatment will be possible. Moreover, the findings of the drug sensitivity analysis of chemotherapeutic agents, targeted agents and immunotherapeutic agents also provide a reference for the rational choice of HCC treatment. More importantly, regarding the evaluation and prediction of immunotherapy efficacy, researchers found that the TIDE score was the best predictor of immune checkpoint inhibitor therapy compared to all candidate biomarkers as well as two single indicators of dysfunction and rejection as reported in the literature [18]. Based on the scoring results presented here, the high TIDE scores in the high-risk group strongly indicated that an increase in risk was also accompanied by increased resistance to immunotherapy to the extent that it might be associated with lower patient survival under ICI treatment.

However, some limitations associated with this study should be noted. First, all analyses were performed by mining public databases. Although we validated of the expression levels of lncRNAs associated with model construction, studies on their mechanistic roles and associated phenotypes are lacking. Moreover, practical application of our model remains an issue that needs to be addressed, emphasizing the desperate need for in-depth work in the real world.

## Conclusion

In summary, our series of comprehensive analyses contributed to the development of a valuable signature that exhibited promising prognostic performance and strongly correlated with immune cell infiltration characteristics and TMB. This signature will provide us with in-depth biological insights to facilitate the identification of prognostically and therapeutically relevant biomarkers for HCC.

## Supplementary Information

**Additional file 1.**

## Data Availability

The datasets analysed during the current study are available in the TCGA (https://gdc.cancer.gov/) and Matrisome Project (www.matrisomeproject.mit.edu/other-resources/human-matrisome) repositories.

## Abbreviations

HCC: Hepatocellular Carcinoma
ECM: Extracellular Matrix
LASSO: Least Absolute Selection and Shrinkage Operator
TMB: Tumour Mutation Burden
ECMrlncRNAs: ECM-related LncRNAs
RNA-seq: RNA Sequencing
HR: Hazard Ratios
PCA: Principal Component Analysis
OS: Overall Survival
DEGs: Differentially Expressed Genes
ICIs: Immune Checkpoint Inhibitors
MSM: Masked Somatic Mutation
TME: Tumour Microenvironment
CTLA-4: Cytotoxic T Lymphocyte Protein 4
