# Supplementary material for: Development and validation of an ECM-related prognostic signature to predict the immune landscape of human hepatocellular carcinoma
Source: BMC Cancer. 2022 Oct 4;22:1036. doi: 10.1186/s12885-022-10049-w (PMC9531523; doi:10.1186/s12885-022-10049-w)
Supplement: Supplementary file 1 — Additional file 1. [file 12885_2022_10049_MOESM1_ESM.docx]

**Figure legend**


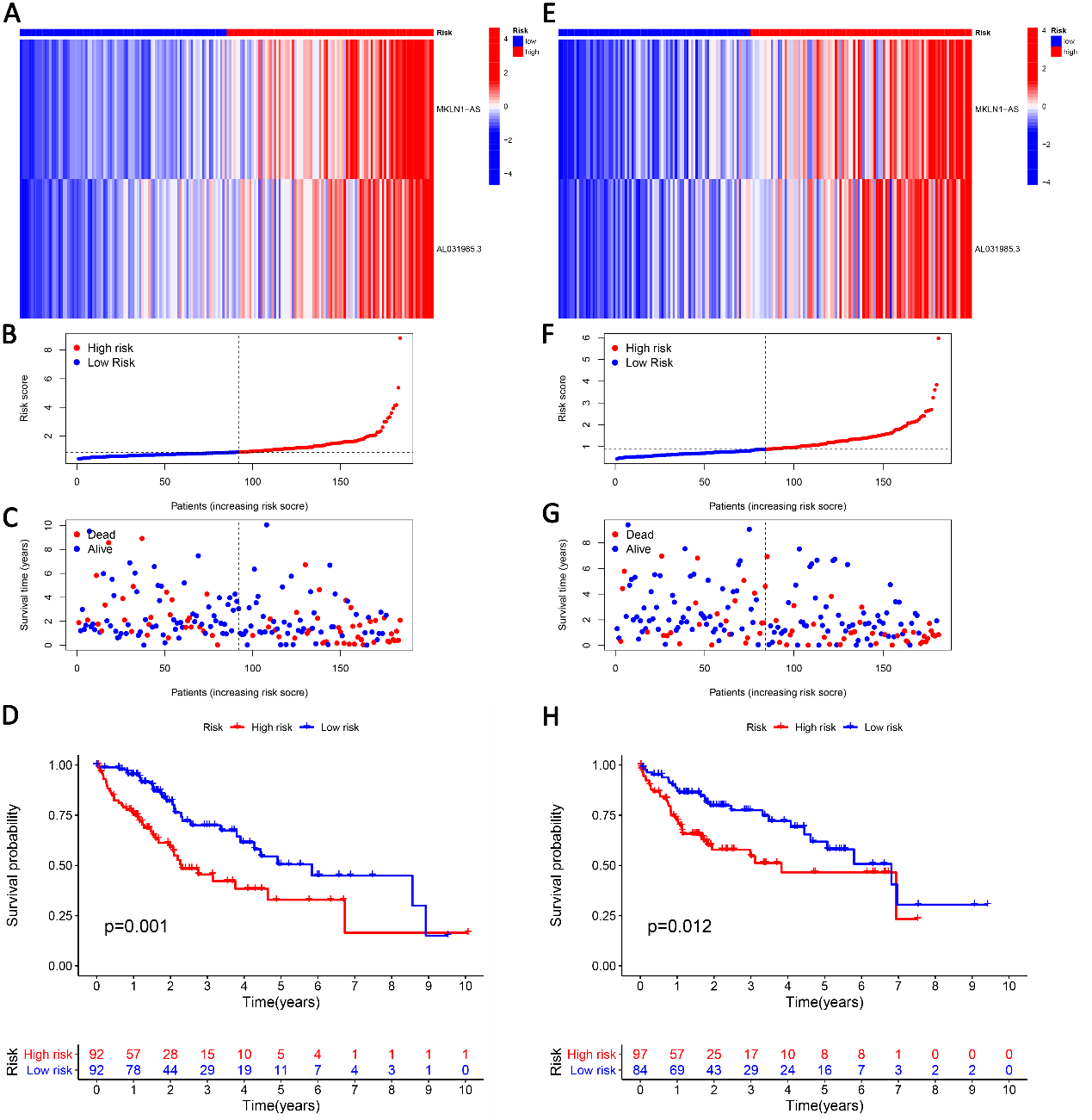


**Figure S1** **Identification of Prognosis-Related ECMrlncRNAs and Signature Development.** Heatmap visualizing the expression levels of MKLN1-AS and AL031985.3 in the training (A) and testing cohort (E). PCA displays the distribution of different-risk patients and differences in survival status (alive or dead) in the training cohort (B and C) and testing cohort (F and G). Survival curves revealed the prognostic differences between the high- and low-risk HCC groups in the training cohort (D) and testing cohort (H)
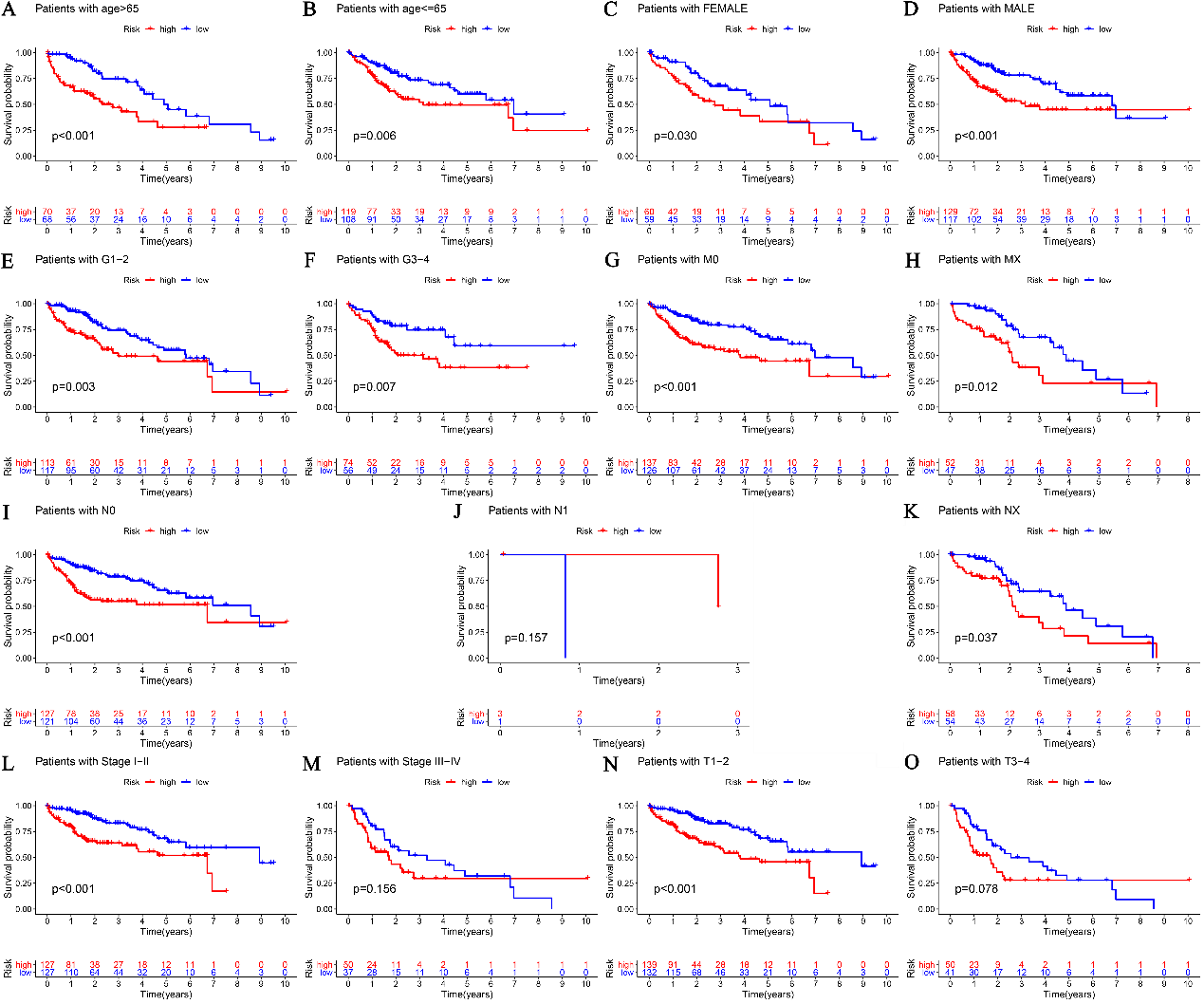


**Figure S2** **Exploration of the Correlation between the Signature and Clinicopathological traits.**

Significant differences between the high- and low-risk groups were identified when patients were stratified by age (A and B), sex (C and D), grade (E and F) and TNM stage (G-I and K-O). However, no significant differences were noted based on stratification analysis by N1 stage (J)
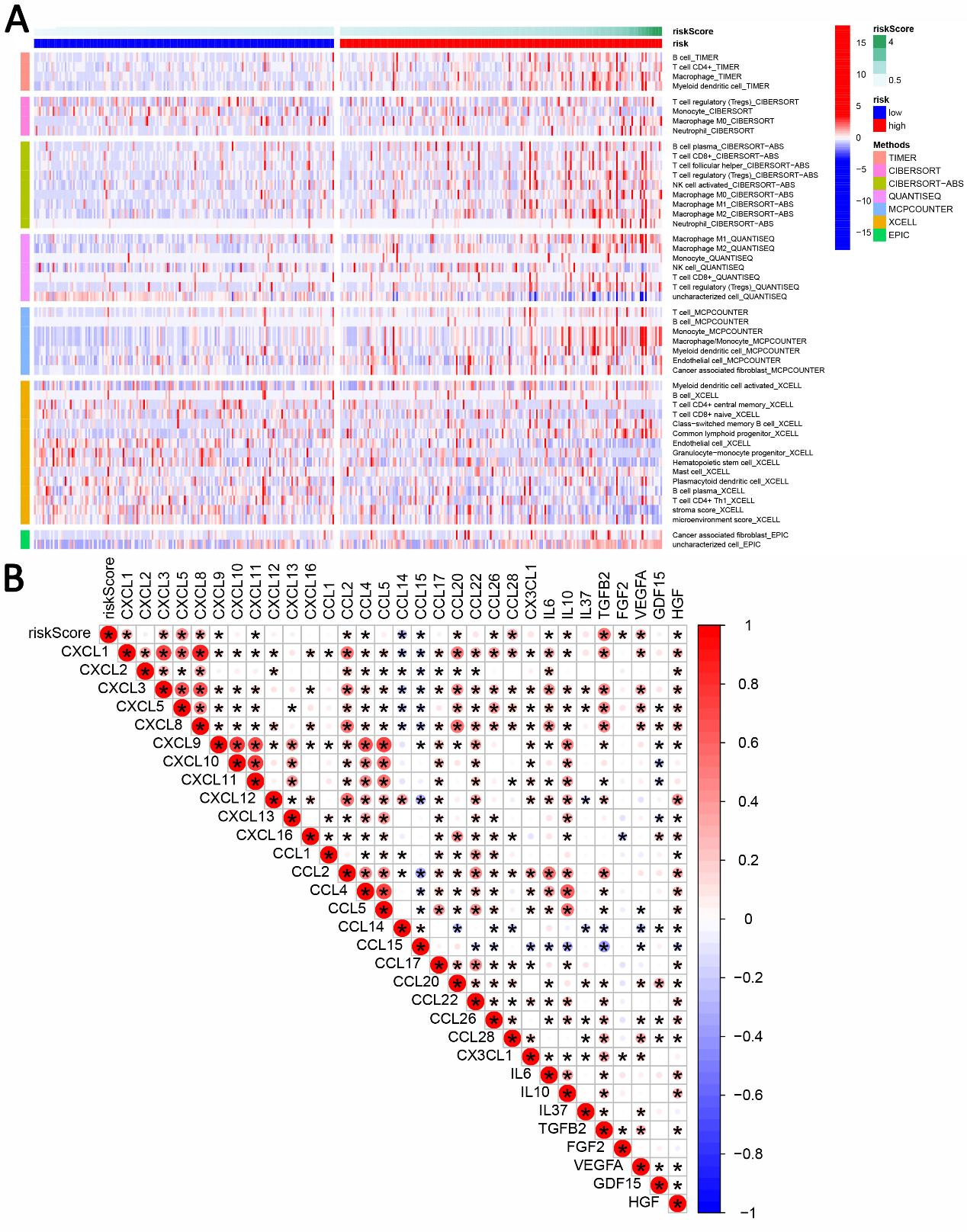


**Figure S3 Correlation Analysis of Risk Scores With Cytokines and Chemokines.**

The differences in tumour-infiltrating immune cells between the two groups are presented as a heatmap using the TIMER, CIBERSORT, CIBERSOT-ABS, QUANTISEQ, MCPCOUNTER and XCELL algorithms (A). The relationship between the risk score and cytokines and chemokines is shown in Panel (B). Asterisks indicate significant correlations. Red represents a positive correlation, and blue represents a negative correlation
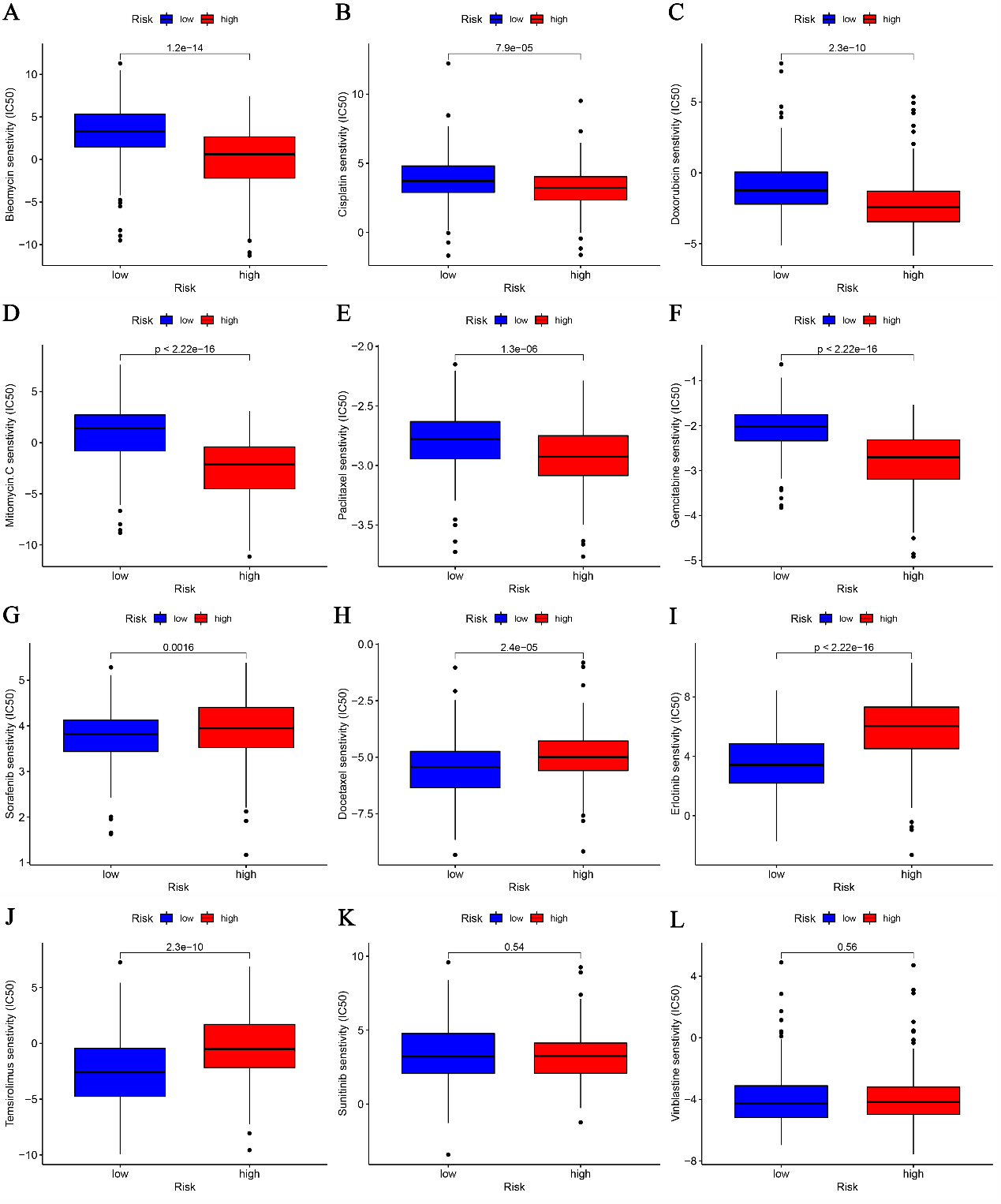


**Figure S4** **Drug Sensitivity Analysis.**

The differences in drug sensitivity to chemotherapeutic agents and targeted agents between the two risk groups are presented as boxplots (A-L)
